# Supplementary material for: A shape-based functional index for objective assessment of pediatric motor function
Source: PLoS One. 2025 Oct 10;20(10):e0332383. doi: 10.1371/journal.pone.0332383 (PMC12513642; doi:10.1371/journal.pone.0332383)
Supplement: Attachment — Submitted filename: Response_to_Reviewers.docx [file pone.0332383.s001.docx]

**Response to Editor:**

We thank the editor for the suggestions which were extremely helpful for strengthening the paper. We have modified our template with PLOS One’s latex template to address requirements 1 and 3. We are sharing the code at [GITHUB LINK](https://github.com/BarnesLab/UExtendPLOS/blob/main/paper_codes.ipynb) to address point 2. We have removed the funding information from the manuscript to address point 4. A complete anonymized version of the data has been hosted at <https://zenodo.org/records/16757630> to address point 5 and 6. ORCID has been provided for point 7 and images have been removed from manuscript for point 8. Finally, we have added the revised manuscript with track changes (changed text in blue text and changed figure in blue caption) and an unmarked version of our revised paper with this letter.

**Response to Reviewers:**

We thank the reviewers for their constructive suggestions. Both the reviews are extremely well thought, and we appreciate the reviewers for also providing explicit suggestions on how to address the reviews.

**Reviewer #1:**.

***Specific Comments:***

***1. Insufficient Discussion on FDA Approval of MetaMotionR+:*** *The study employs MetaMotionR+, a wearable sensor primarily designed for research and development, which has not been approved by the U.S. Food and Drug Administration (FDA). Given that the manuscript suggests the potential clinical application of this method, the absence of discussion regarding FDA approval and regulatory considerations is a notable gap. Including the following considerations would strengthen the manuscript:*

*a. If this method is to be implemented as a medical device, FDA approval may be required.*

*b. MetaMotionR+ is currently a research-grade device, and stricter regulatory compliance would be necessary for its use as a clinical evaluation tool.*

*c. Future expansion of clinical trials and steps toward obtaining FDA approval should be discussed.*

*Without such considerations, the study remains a research-level proposal and does not sufficiently address the challenges of actual clinical implementation. If clinical application is indeed envisioned, discussion of the medical device approval process and current limitations of the technology should be incorporated.*

**Response:** Thank you for raising this important point and we understand your concern. We have added an acknowledgement of the fact that MetaMotionR+ is not yet FDA approved in our discussion section. That being said, the Shape Principal Component Analysis technique is agnostic to choice of wearable sensor and there are several consumer grade wearables with FDA approval (e.g., the Actigraph Leap and Empatica Embrace) which could be viable alternatives for implementing this index as a medical device. We have added the following statement on lines 335-338 to address this.

**Although we used the MetaMotionR+ sensor, which is not FDA-approved, the Shape PCA approach is sensor-agnostic and compatible with consumer-grade wearables. Several FDA-approved alternatives (e.g., Actigraph LEAP [41], Empatica Embrace [42]) may be viable for clinical implementation.**

***2. Lack of Discussion on Why Certain Movements Did Not Yield Clear Results:*** *The study analyzed five types of movements: Rotating a doorknob, Raising a cup, Arm curl, Door knocking, Moving a paddle. Among these, VPC1 Curl (arm curl speed) and VPC1 Knock (knocking motion speed) exhibited clear differences in motor function and were analyzed in detail. However, the doorknob rotation, cup lifting, and paddle movement were also subjected to Shape PCA, but their principal components were difficult to interpret, and no conclusive results were obtained. Despite this observation, the manuscript does not sufficiently discuss why these movements did not yield clear findings. Possible explanations include:*

*a. These movements may exhibit significant individual variation, making it difficult to derive unified principal components via PCA.*

*b. Variability in movement initiation and termination may have interfered with Curve Registration, affecting alignment accuracy.*

*c. These movements may be less influenced by muscle strength, leading to weaker differentiation among disease groups.*

*d. A wrist-worn sensor alone may not have been sufficient to capture these movements accurately.*

*e. These tasks may not adequately reflect key features of DMD and SMA, such as muscle weakness and movement asymmetry.*

*Since the manuscript does not address these possibilities, expanding the discussion on why only arm curl and door knocking produced interpretable results, while other movements did not, would enhance the study’s credibility.*

*Addressing these points would further enhance the manuscript’s clinical and scientific relevance, increasing its overall impact and robustness.*

**Response:** We believe the reviewer has already covered most of the potential explanations related to this point. This is indeed what we observed. The Shape PCA modes for other activities are a lot noisier than the ones for curl or knock. We have added a potential explanation of this phenomena in discussion lines 322-330 and copied below. Since Shape PCA seeks to maximize variance instead of optimizing for separability of different classes, we do not believe that should affect noise levels of Shape Principal Components.

**While we examined multiple movements, not all yielded interpretable Shape PCA modes and were therefore excluded from the motor function index. Several factors may explain this. First, movements with high inter-individual variability may require a larger sample size to reliably estimate the covariance structure in Shape PCA. Additionally, certain tasks (e.g., rotating a doorknob or moving a paddle) produced noisier data than others, potentially due to their greater difficulty for participants compared to simpler actions like the arm curl. It is also possible that a single wearable sensor lacks the resolution to capture subtler movements, leading to challenges in segmentation and registration.**

**Reviewer #2:**

***Specific Comments:***

***1. Test-Retest Reliability, Consistency of Index over multiple activities:*** *There are, however, a few areas that would benefit from further clarification or elaboration. The paper does not include any analysis of test-retest reliability or discussion of how consistent the Shape-based index would be if measured over time or across different daily activities. While the current findings are compelling, the stability and reproducibility of the index should be discussed more explicitly.*

**Response:** Thank you for raising this important point. Usually one would perform a test-retest analysis if the data can be collected by different instruments (locations) over different times. However, our data was collected by the same sensor in a single study performed at a fixed location. As stated in the section on statistical analysis, since we randomly sample a visit per participant to ensure IID (Independent and Identically Distributed) assumption of PCA, the bootstrap distribution provides us with an estimate of variability across visits. We also provide group level analysis on trends across cohorts. Unfortunately, due to the rare nature of this disease, we currently do not have the requisite number of longitudinal visits and trials per single participant to be able to monitor the stability of the index at a participant level over time. We have added the following statement to acknowledge this limitation in lines 338-341 of discussion.

**Finally, due to limited repeat visits, we focused on cross-sectional analysis; future work will aim to increase follow-up data to examine longitudinal changes in motor function, including progression, stability, or regression, in relation to disease course and treatment response.**

Regarding the second part of your comment — the stability of the index across different activities — we would appreciate clarification on what is meant by test-retest reliability in this context. Our analysis treats each activity (e.g., arm curl, knock) separately, producing distinct biomarkers (VPC coefficients) for each. As we report in line 261 of our manuscript, the proposed index is a linear combination of multiple features, 0.76*speed of curl - 0.59*speed of knock + 0.18*asymmetry in curl + 0.18*asymmetry in knock. In Fig 9 (also pasted below), we show the distribution of coefficients for each of these features. As we can see in Fig 9, the features with higher weightage (e.g., the speed features) also have lesser variability in their coefficient distribution. Thus the distribution plots provide a sense of how important each activity is to the index and shows the consistency of our motion index because of its narrow distribution of first canonical correlation coefficient and less variability.

***2. Participant Exclusion:*** *Another point to consider is the potential bias introduced by the exclusion of certain participants—particularly those who were unable to complete tasks due to low function or technical issues—which may limit the generalizability of the results. A brief acknowledgment of this limitation would strengthen the transparency of the study.*

**Response:** This is an excellent point, and we have added the following statement in discussion lines 331-334 to acknowledge this limitation.

**We also excluded some younger participants who were unable to follow complex instructions, which may limit the generalizability of our findings. To address these issues, we are refining our study design to focus on movements that are easier for most participants to perform-arm curl and knock, in particular, showed strong potential.**

***3. Data sharing****: Although a reduced version of the dataset and the code are made available via GitHub, and this inclusion is appreciated, the full underlying data are only available upon request. In accordance with the journal’s data policy, it is recommended to provide more detailed summary-level data or anonymized datasets when possible or to more clearly justify any restrictions.*

**Response:** Thank you, this is a crucial point. In order to improve replicability, we are releasing the full anonymized version of the dataset here <https://zenodo.org/records/16757630>.

***4. Aesthetic Changes:*** *Some visual elements, such as the vertical principal component plots, could benefit from clearer labeling or interpretive annotations to guide the reader—especially those from a clinical background. Enhancing figure legends or adding simple indicators of directionality (e.g., faster/slower motion) could improve interpretability. Lastly, there are a few minor issues related to language and formatting, such as inconsistent use of "Fig." vs. "Figure" and some long or complex sentences that could be streamlined.*

**Response:** We have added labels for the VPC modes in Fig 5 as shown below. While we were unable to find instances of “Figure” in our text, we have tried to simplify the complex sentences. We have also pluralized Figs for multiple instances of figures in order to be consistent with PLOS One’s policy.

***Summary Comment:*** *In conclusion, this paper is a well-structured and valuable contribution. Addressing the above suggestions would enhance the clarity, reproducibility, and practical applicability of the work and can be achieved without the need for additional experiments.*

**Response:** Thank you for your positive assessment and constructive feedback. We appreciate your recognition that our work represents a valuable contribution to the field. We have carefully addressed your suggestions in our revision to discuss limitations and enhance the reproducibility, and practical applicability of our findings.
